# Haematological and Biochemical Reference Values for Healthy Adults in the Middle Belt of Ghana

**DOI:** 10.1371/journal.pone.0036308

**Published:** 2012-04-27

**Authors:** David K. Dosoo, Kingsley Kayan, Dennis Adu-Gyasi, Evans Kwara, Josephine Ocran, Kingsley Osei-Kwakye, Emmanuel Mahama, Stephen Amenga-Etego, Philip Bilson, Kwaku P. Asante, Kwadwo A. Koram, Seth Owusu-Agyei

**Affiliations:** 1 Kintampo Health Research Centre, Kintampo, Ghana; 2 Noguchi Memorial Institute for Medical Research, University of Ghana, Legon, Ghana; Lund University Hospital, Sweden

## Abstract

**Background:**

Reference values are very important in clinical management of patients, screening participants for enrolment into clinical trials and for monitoring the onset of adverse events during these trials. The aim of this was to establish gender-specific haematological and biochemical reference values for healthy adults in the central part of Ghana.

**Methods:**

A total of 691 adults between 18 and 59 years resident in the Kintampo North Municipality and South District in the central part of Ghana were randomly selected using the Kintampo Health and Demographic Surveillance System and enrolled in this cross-sectional survey. Out of these, 625 adults made up of 316 males and 309 females were assessed by a clinician to be healthy. Median values and nonparametric 95% reference values for 16 haematology and 22 biochemistry parameters were determined for this population based on the Clinical Laboratory and Standards Institute guidelines. Values established in this study were compared with the Caucasian values being used currently by our laboratory as reference values and also with data from other African and western countries.

**Results:**

Reference values established include: haemoglobin 113–164 g/L for males and 88–144 g/L for females; total white blood cell count 3.4–9.2×10^9^/L; platelet count 88–352×10^9^/L for males and 89–403×10^9^/L for females; alanine aminotransferase 8–54 U/L for males and 6–51 U/L for females; creatinine 56–119 µmol/L for males and 53–106 µmol/L for females. Using the haematological reference values based on the package inserts would have screened out up to 53% of potential trial participants and up to 25% of the population using the biochemical parameters.

**Conclusion:**

We have established a panel of locally relevant reference parameters for commonly used haematological and biochemical tests. This is important as it will help in the interpretation of laboratory results both for clinical management of patients and safety monitoring during a trial.

## Introduction

Locally relevant reference ranges for commonly used biochemical and haematological parameters are essential for screening and safety follow up of trial participants as well as for routine clinical management of patients. However, reference values being used in most laboratories in African countries have been obtained from the literature, reagent inserts accompanying the reagent kits or instrument manuals [1]. These values more often than not have been derived from Caucasian populations of industrialised countries and may not be applicable in most local settings. Factors such as age, gender [2], ethnicity [3] and environment including altitude and geo-chemicals [4] affect the measurements determined in different populations. Published literature has confirmed that many of the reference values obtained from the developed countries differ significantly from what pertains in most African localities [1], [5], [6], [7], [8]; thus making it necessary to establish locally relevant values. The Clinical and Laboratory Standards Institute (CLSI) [9] and the International Federation for Clinical Chemistry (IFCC) [10] recommend that each laboratory establishes its own reference values.

The Kintampo Health Research Centre (KHRC) located in Central Ghana has been undertaking Phase II/III drugs and vaccines trials since 2003 and plans soon to add Phase I trials in infectious diseases. Locally relevant biochemical and haematological reference values for the population are needed in determining eligibility of participants being enrolled into future studies and also for monitoring of the onset of any adverse events during a trial. Availability of such reference values in the locality would also assist physicians in the management of patients. This study was, thus, aimed at establishing reference values for commonly used haematological and biochemical parameters in the population within the Kintampo North Municipality and Kintampo South District.

## Methods

### Study Site

**Figure 1:**
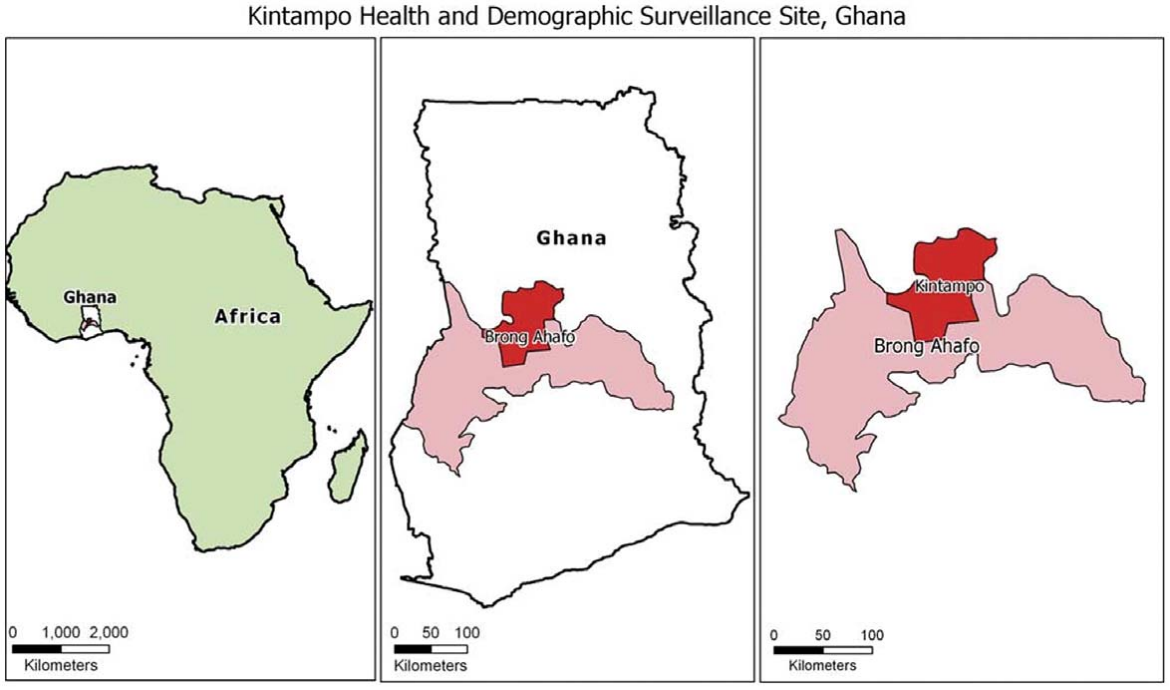
Map of the study area.

**Figure 2:**
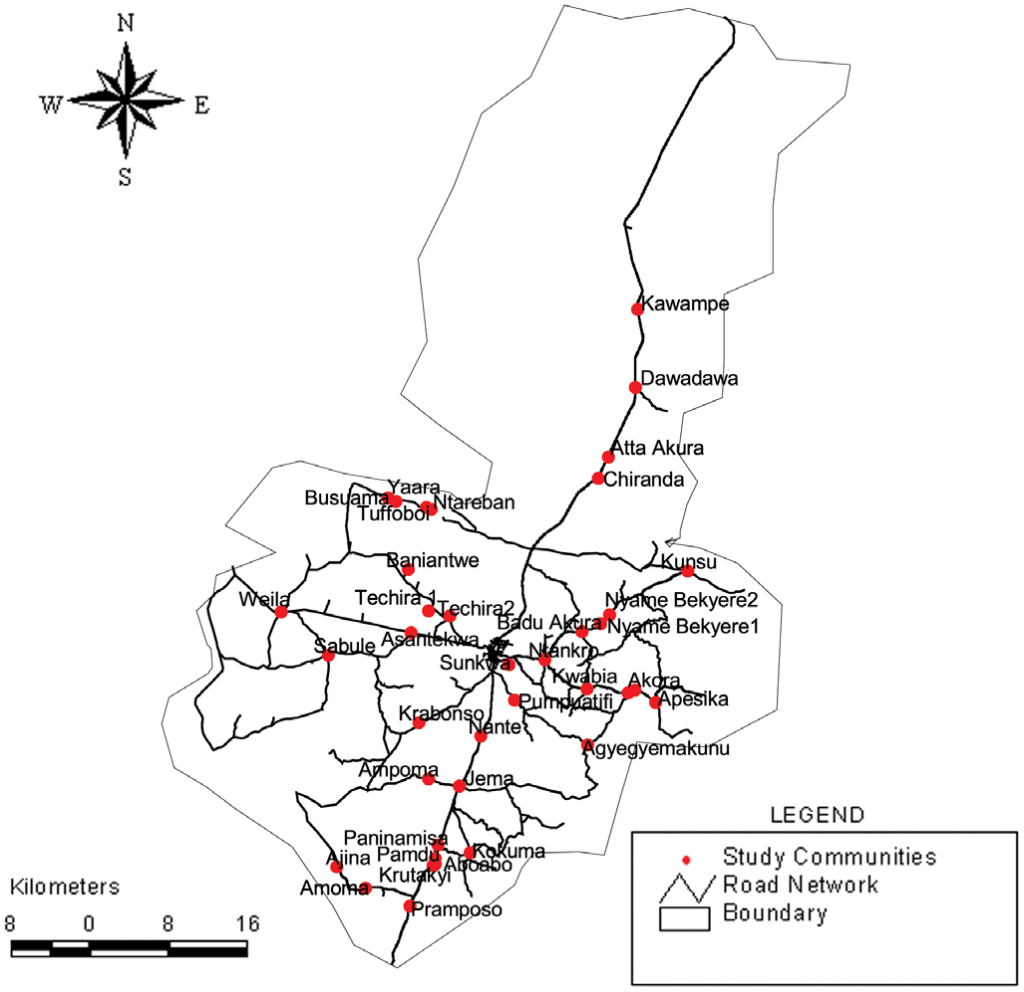
Map of study communities.

The study was carried out in the Kintampo North Municipality and Kintampo South District of the Brong Ahafo Region of Ghana (Figure 1 and Figure 2). The studied area is located between Latitudes 7°43′N and 8°44′N and Longitudes 1°25′W and 2°1′W. It lies within the forest-savannah transitional ecological zone and has an elevation ranging between 60 and 150 m above sea level. It is made up of a resident population of about 140,000. The Kintampo Health Research Centre maintains a Health and Demographic Surveillance System (HDSS) that records detailed demographics of all residents including pregnancies, births, deaths and migrations (in and out) at 4 monthly intervals. All the compounds have been digitized making the selection and tracing of individuals to their homes easy.

### Selection of reference population

The communities and individuals who participated in this study were randomly selected from the HDSS human population using the Visual FoxPro software. Community meetings were held to explain the objectives of the study to the opinion leaders and other community members.

Those selected through the randomisation were invited to a central location where individual consenting, screening and blood collections were carried out. Inclusion into the study was based on willingness of the individual to participate in the study (demonstrated by the completion and signing/thumbprinting of the consent form and willingness to provide the samples required), general good health (as determined by a clinician's medical history and physical examination, and residence in the study area for at least 3 months. Individuals with evidence of acute or chronic respiratory, cardiovascular, gastrointestinal, hepatic or genitourinary conditions, history of blood donation/transfusion within the immediate past three months, hospitalisation within the immediate past one month, or any other findings that in the opinion of the examining clinician may compromise on the assessment of the laboratory parameters of interest in this study were excluded. Those assessed to be pregnant (either clinically or by positive urine β-HCG test) and lactating mothers were all excluded.

### Laboratory analysis

Venous blood samples were collected from the antecubital fossa, dispensed into a 2 ml K_3_EDTA, a 5 ml SST tubes with gel and 1 ml Fluoride-EDTA for haematology, biochemistry and glucose analysis, respectively. Sample tubes were from Becton Dickinson (Plymouth, United Kingdom). Haematological analysis (complete blood count with 3-part differential) was performed using previously validated ABX Micros 60 analysers (Horiba-ABX, Montpellier, France). Calibrators and controls were obtained from the instrument manufacturer. Analysis of samples was performed within 8 hours of blood draw.

Samples for biochemical analysis were allowed to clot for at least 60 minutes, centrifuged and the serum collected. Serum was analysed within 24 hours after collection. If testing was delayed, serum was stored frozen at −80°C and subjected to a single freeze-thaw cycle at the time of analysis. The Vitalab Selectra E Clinical Chemistry analyser (Vital Scientific, The Netherlands) was used to perform the analysis. Test tubes for the clinical chemistry analysis were from Vital Scientific, The Netherlands. Reagents, calibrators and controls were from Elitech Diagnostics (Sees, France). Electrolytes (chloride, potassium and sodium) were analysed using the Humalyte Electrolyte analyser (Human Diagnostics, Germany). Reagents were from the manufacturer of the instrument.

Normal and abnormal controls were run daily. No analysis was done if controls were out of range. In addition to the internal quality assessment, the laboratory participates in external quality assessments for haematology and clinical chemistry both with the College of American Pathologists (CAP) and the United Kingdom National External Quality Assessment Scheme (UK NEQAS). The laboratory complies with the principles of Good Clinical Laboratory Practice [11], [12]. Individuals with abnormal clinical or laboratory test results were referred for appropriate care and treatment.

Between-run precision for the analytes were assessed using at least 20 measurements made on separate days using normal control samples. The mean, standard deviation (SD) and coefficient of variation (CV) were calculated for each analyte. Coefficients of variation (CVs) were compared to those quoted in the analyser manuals and reagent inserts.

### Data Management and Statistical Analysis

Data were recorded on questionnaires, double-entered into a Visual FoxPro 9.0 database and verified. Data analysis was carried out using Stata 11 (Stata Corp, College Park, TX, USA). The 2.5^th^ and 97.5^th^ percentiles were determined non-parametrically. This was according to the CLSI/IFCC guidelines on defining, establishing and verifying reference intervals in the clinical laboratory [9]. To obtain these intervals, a minimum of 120 observations were required for each analyte within each subgroup. Outliers within each subgroup were identified using the Dixon method [9]. Briefly, the extreme values were retained in the distribution if D/R<0.33, where D is the absolute difference between the most extreme distribution and the next value and R is the Range (maximum – minimum). Reference values were determined separately for males, females and combined gender. Differences between genders were tested using the Mann-Whitney test. The values defined were compared with the recommended reference values (based on a North American population) provided in the ABX Micros 60 Haematology User Manual [13] and Elitech Diagnostics chemistry reagent inserts, respectively.

### Ethical Considerations

This study was approved by Ethics Committees of the Kintampo Health Research Centre, the Noguchi Memorial Institute for Medical Research and the Ghana Health Service. Written informed consent was obtained from each participant prior to involving them in the study.

## Results

A total of 691 randomly selected adults made up of 351 males and 340 females between the ages of 18 and 59 years (mean = 37 years) were screened during the survey. Out of this, 625 individuals (316 males and 309 females) with a mean age of 36 years were enrolled. Tobacco use among all males screened was 64 (9.26%) out of which 52 (8.3%) were among those enrolled. None of the females screened and/or enrolled was a smoker. Screened and enrolled males and females who take alcohol were 107 (33.75%) and 34 (10.97%) respectively. Forty-three (6.2%) of the screened population were taking prescribed medication and were excluded from the study.

### Reference Values

Median and 95% reference values (2.5^th^–97.5^th^ percentiles) for Haematology and Biochemistry are shown in Tables 1 and 2, respectively. Males had significantly higher haemoglobin values of 113–164 against 88–144 g/L for females (p<0.0001), haematocrit of 33.2–50.5 against 26.4–45.0% (p<0.0001) and red blood cells (RBC) of 3.79–5.96 against 3.09–5.30×10^12^/L (p<0.0001) compared to females. On the other hand, platelets were significantly higher in females with 89–403 against 88–352×10^9^/L for males (p<0.0001). For the biochemical values, males had significantly higher alanine aminotransferase (ALT) of 8–54 against 6–51 U/L (p<0.0001), aspartate aminotransferase (AST) of 17–60 against 13–48 U/L (p<0.0001), alkaline phosphatase (ALP) of 101–353 against 82–293 U/L (p<0.0001), Creatine kinase (CK) of 93–786 against 58–476 U/L (p<0.0001), gamma glutamyltransferase (GGT) of 9–71 against 6–53 U/L (p<0.0001), Creatinine of 56–119 against 47–110 µmol/L (p<0.0001), Iron of 6.0–32.8 against 5.4–27.8 µmol/L (p<0.0001), and Uric acid of 126–418 against 83–381 µmol/L (p<0.0001), compared to the females. Females, however, had significantly higher Cholesterol levels of 2.1–5.6 against 1.8–5.6 mmol/L for males (p<0.0001).

**Table 1:** Haematology reference values for Kintampo.

Analyte	Unit	Males			Females			Combined males and Females			
		N	Med	Reference Values	N	Med	Reference Values	N	Med	Reference Values	p-value*
Haemoglobin	g/L	316	139	113–164	308	123	88–144	624	131	98–160	<0.0001
Haematocrit	%	316	42.2	33.2–50.5	309	36.9	26.4–45.0	625	39.4	28.9–48.7	<0.0001
RBC	×10^12^	316	4.84	3.79–5.96	307	4.32	3.09–5.30	623	4.57	3.39–5.83	<0.0001
MCV	fl	316	88	70–98	309	86	73–96	625	87	72–97	0.0192
MCH	pg	316	29.1	22.7–33.5	307	28.4	22.3–33.6	623	28.6	22.6–33.5	0.1448
MCHC	g/dL	315	33.1	30.6–36.0	305	33.1	30.4–36.5	620	33.1	30.5–36.2	0.3621
RDW	%	316	13.6	11.5–16.7	309	13.4	11.4–16.8	625	13.5	11.5–16.7	0.2064
Platelets	×10^9^/L	316	208	88–352	309	224	89–403	625	216	89–380	<0.0001
PDW	%	315	15.8	12–23.4	308	15.7	12.6–22.9	623	15.7	12.6–23.0	0.5511
WBC, Total	×10^9^/L	311	5.5	3.5–9.2	309	5.3	3.4–9.3	620	5.4	3.4–9.2	0.0218
Lymphocytes	%	315	41	24.0–57.2	309	42.3	26.9–58.3	624	41.6	25.2–57.7	0.0430
Lymphocytes	×10^9^/L	316	2.2	1.2–5.2	308	2.1	1.2–4.4	624	2.1	1.2–4.4	0.2046
Monocytes	%	316	9.5	5.7–17.4	309	8.4	5.0–14.4	625	8.9	5.3–16.3	<0.0001
Monocytes	×10^9^/L	316	0.5	0.2–1.4	308	0.4	0.2–0.9	624	0.4	0.2–1.0	0.0002
Granulocytes	%	316	48.6	30.2–69.9	309	48.5	33.3–67.5	625	48.5	32.0–68.1	0.8643
Granulocytes	×10^9^/L	313	2.7	1.5–5.9	309	2.7	1.4–5.5	622	2.7	1.5–5.6	0.1943

Med: Median.

* Mann-Whitney test for differences in between males and females.

**Table 2:** Biochemistry reference values for Kintampo.

Analyte (Unit)	Males			Females			Combined males and Females			
	N	Med	Reference Values	N	Med	Reference Values	N	Med	Reference Values	p-value*
Enzymes										
ALT (U/L)	303	23	8–54	300	17	6–51	603	20	7–51	<0.0001
AST (U/L)	300	30	17–60	288	23	13–48	588	26	14–51	<0.0001
ALP (U/L)	293	178	101–353	302	155	82–293	595	160	85–241	<0.0001
Amylase (U/L)	313	69	34–137	308	66	30–139	621	67	32–139	0.0089
Creatine Kinase (U/L)	302	249	93–786	288	165	58–476	590	190	66–532	<0.0001
GGT (U/L)	302	21	9–71	298	16	6–53	600	19	7–61	<0.0001
LDH (U/L)	275	421	274–745	248	389	214–688	523	406	223–723	0.0002
Serum Proteins										
Protein, Total (g/L)	296	72.5	46.7–86.4	282	73.4	55.2–86.9	578	72.9	50.6–86.7	0.1253
Albumin (g/L)	315	42.5	32.7–49.8	307	42.2	33.5–50.4	622	42.3	33.0–49.9	0.7558
Metabolism										
Bilirubin, Total (µmol/L)	310	11.3	3.8–32.0	305	9.4	2.7–26.6	605	9.9	2.9–25.8	0.0006
Bilirubin, Direct (µmol/L)	189	2.9	0.9–4.1	213	2.4	0.7–3.8	402	2.6	0.8–4.0	<0.0001
Cholesterol (mmol/L)	315	3.2	1.8–5.0	307	3.5	2.1–5.6	622	3.3	2.0–5.4	<0.0001
Glucose (mmol/L)	315	4.8	3.5–6.3	307	4.8	3.7–6.6	622	4.8	3.6–6.4	0.5790
Iron (µmol/L)	309	15.0	6.0–32.8	296	13.5	5.4–27.8	605	14.1	5.5–30.6	0.0004
Triglycerides (mmol/L)	314	0.9	0.4–2.2	305	0.9	0.4–2.1	619	0.9	0.4–2.2	0.1388
Kidney Function										
Urea (mmol/L)	315	2.7	0.9–6.2	307	2.5	0.9–5.4	622	2.5	0.9–5.7	0.0066
Creatinine (µmol/L)	312	85	56–119	303	74	47–110	615	80	49–118	<0.0001
Uric Acid (µmol/L)	314	243	126–418	306	181	83–381	620	216	91–399	<0.0001
Serum Electrolytes										
Chloride (mmol/l)	271	107	101–115	260	108	113	531	107	102–114	0.1609
Phosphorus (mmol/L)	315	1.1	0.7–1.5	307	1.1	0.8–1.5	622	1.1	0.7–1.5	0.0202
Potassium (mmol/L)	295	4.5	3.6–5.2	288	4.3	3.4–5.1	583	4.4	3.6–5.2	<0.0001
Sodium (mmol/L)	276	144	135–151	265	145	135–150	541	144	135–150	0.1080

Med: Median ; NA: Not available.

* Mann-Whitney test for differences between males and females.

The column percentage out-of range (% OOR) in Tables 3 and 4 shows the proportion of normal adults in the Kintampo area who would have been classified as having abnormal haematology and biochemistry results when compared with the recommended reference values (based on a north American population) provided in the ABX Micros 60 Haematology User Manual [13] and Elitech Diagnostics reagent inserts, respectively. Out of range values for haematology ranged between 13 and 96% with parameters such as Haemoglobin, Haematocrit, RBC, mean cell volume (MCV), mean cell haemoglobin (MCH), mean cell haemoglobin concentration (MCHC) and white blood cell (WBC) predominantly on the lower side of the comparison values. OOR values for biochemistry were high for Amylase (28% and 18%), CK (74% and 58%), lactate dehydrogenase (LDH) (37% and 28%), Protein (34% and 31%), Total Bilirubin (23% each), Urea (25% and 32%), Uric Acid (30% and 36%) and Phosphorus (23% and 24%) for males and females, respectively. Tables 5 and 6 show a comparison of haematology and biochemistry reference values established for Kintampo with values from other studies. Between-run precision for the haematology and clinical chemistry assays are presented in Tables 7 and 8, respectively. The methods used for the various clinical chemistry analytes are also shown in Table 8.

**Table 3:** Haematology out of range (OOR) values based on comparison with ABX values.

Analyte	Unit	Males		Females	
		ABX Values	% OOR	ABX Values	% OOR
Haemoglobin	g/L	135–165	37.0	120–150	42.6
Haematocrit	%	41–50	41.6	37–45	53.1
RBC	×10^12^	4.37–5.63	26.6	3.9–5.10	27.7
MCV	fl	83–101	23.7	84–94	39.5
MCH	pg	26–34	14.3	27–34	25.5
MCHC	g/dL	32–35	26.4	32–35	31.8
RDW	%	12–16	13.6	12–14	43.7
Platelets	×10^9^/L	145–355	16.1	150–330	20.1
PDW	%	NA	NA	NA	NA
WBC, Total	×10^9^/L	4.7–9.6	26.1	4.9–12.3	38.9
Lymphocytes	%	23–47	22.9	19–41	56.3
Lymphocytes	×10^9^/L	NA	NA	NA	NA
Monocytes	%	3–6	95.9	2–6	90.1
Monocytes	×10^9^/L	NA	NA	NA	NA
Granulocytes	%	49–74	52.2	53–79	68.6
Granulocytes	×10^9^/L	NA	NA	NA	NA

NA: Not available.

**Table 4:** Biochemistry out of range (OOR) values based on comparison with values from Elitech reagent inserts.

Analyte (Unit)	Males		Females	
	Elitech Values	% OOR	Elitech Values	% OOR
Enzymes				
ALT (U/L)	0–40	11.8	0–40	5.6
AST (U/L)	0–46	14.9	0–46	5.2
ALP (U/L)	0–270	16.6	0–240	10.8
Amylase (U/L)	0–90	28.0	0–90	18.2
Creatine Kinase (U/L)	0–171	74.2	0–145	58.3
GGT (U/L)	0–55	11.4	0–38	4.9
LDH (U/L)	235–470	36.6	235–470	27.6
Serum Proteins				
Protein, Total (g/L)	60–78	33.8	60–78	31.2
Albumin (g/L)	35–52	4.8	35–52	5.5
Metabolism				
Bilirubin, Total (µmol/L)	5–21	22.7	5–21	22.5
Bilirubin, Direct (µmol/L)	0–4	2.1	0–4	0.5
Cholesterol (mmol/L)	0–5	1.9	0–5	8.1
Glucose (mmol/L)	4.0–6.0	15.3	4.0–6.0	12.0
Iron (µmol/L)	9.0–30.0	16.5	9.0–30.0	16.5
Triglycerides (mmol/L)	0–1.7	6.4	0–1.7	6.2
Kidney Function				
Urea (mmol/L)	2.0–7.0	25.0	2.0–7.0	31.6
Creatinine (µmol/L)	71–115	21.6	53–106	8.9
Uric Acid (µmol/L)	208–428	29.6	155–357	36.3
Serum Electrolytes				
Chloride (mmol/l)	NA	NA	NA	NA
Phosphorus (mmol/L)	0.9–1.5	23.2	0.9–1.5	23.7
Potassium (mmol/L)	NA	NA	NA	NA
Sodium (mmol/L)	NA	NA	NA	NA

NA: Not available.

**Table 5:** Comparison of adult haematological reference values obtain from this study against others.

Analyte	Unit	Present Study	Southern Ghana [1]	Kenya [16]	Eastern & Southern Africa [7]	Mbeya, Tanzania [20]	USA [14]
Haemoglobin (M)	g/L	113–164	117–165	83–113	122–177	137–177	135–175
Haemoglobin (F)	g/L	88–144	91–140	59–100	95–158	111–157	120–160
Haematocrit (M)	%	33.2–50.5	37.1–51.4	40–50	35.0–50.8	40.2–53.7	41.0–53.0
Haematocrit (F)	%	26.4–45.0	29.1–43.6	30–50	29.4–45.4	36.2–46.8	36.0–46.0
RBC (M)	×10^12^/L	3.79–5.96	NA	4.4–6.3	4.0–6.4	4.41–6.27	4.5–5.9
RBC (F)	×10^12^/L	3.09–5.30	NA	3.7–5.6	3.8–5.6	3.84–5.59	4.0–5.2
MCV	fl	72–97	NA	69–97	68–98	78–98	80–100
MCH	pg	22.6–33.5	NA	22.4–33.5	NA	23.6–33.1	26.0–34.0
MCHC	g/dL	30.5–36.2	NA	32.2–33.5	NA	30.6–34.9	31.0–37.0
Platelets (M)	×10^9^/L	88–352	97–356	115–366	150–350	147–356	150–350
Platelets (F)	×10^9^/L	89–403	118–385	124–444	150–350	151–425	150–350
WBC, Total	×10^9^/L	3.4–9.2	3.4–8.8	2.8–8.4	3.1–9.1	3.0–7.9	4.5–11.0

NA, Not Available.

**Table 6:** Comparison of adult biochemical reference values obtained from this study against others.

Analyte	Unit	Present Study	Southern Ghana [1]	Kenya [16]	Tanzania [20]	USA [14]
Sodium	mmol/L	135–150	138–146	141–153	134–143	136–145
Potassium	mmol/L	3.6–5.2	3.1–4.6	3.9–5.8	3.8–5.5	3.5–5.0
Chloride	mmol/L	102–114	NA	101–112	98–107	98–106
Urea	mmol/L	0.9–5.7	1.7–7.2	1.4–4.6	1.5–5.0	3.6–7.1
Creatinine, M	µmol/L	56–119	81–141	62–106	48–96	<133
Creatinine, F	µmol/L	47–110	70–121	51–91	40–81	<133
ALT (M+F)	U/L	7–51	NA	10–52	8–48	0–35
ALT, M	U/L	8–54	12–53	11–54	9–55	0–35
ALT, F	U/L	6–51	10–39	9–47	7–45	0–35
AST, (M+F)	U/L	14–51	NA	14–42	14–48	0–35
AST, M	U/L	17–60	19–65	15–45	15–53	0–35
AST, F	U/L	13–48	16–47	13–38	14–35	0–35
ALP, (M+F)	U/L	85–242	NA	NA	46–158	30–120
ALP, M	U/L	101–353	124–479	NA	45–170	30–120
ALP, F	U/L	82–293	98–316	NA	45–155	30–120
GGT	U/L	7–61	NA	NA	8–108	1–94
Bilirubin, Total	µmol/L	2.9–25.8	1.7–27.0	4.9–39.9	5.2–41.0	5.1–17.0
Bilirubin, Direct	µmol/L	0.8–4.0	3.4–10.3	1.1–8.8	0.7–8.2	1.7–5.1
Albumin	g/dL	33.0–49.9	46–68	35.8–48.1	35.6–50.4	35–55
Total Protein	g/dL	51–87	NA	NA	66–85	55–80
Cholesterol	mmol/L	2.0–5.4	NA	2.6–5.7	2.5–5.5	<5.17
Triglyceride	mmol/L	0.4–2.2	NA	0.4–2.6	0.4–2.9	<1.8
Uric Acid (M)	µmol/L	123–418	NA	NA	196–459	150–480
Uric Acid (F)	µmol/L	83–381	NA	NA	148–360	90–360
Glucose (Fasting)	mmol/L	3.6–6.4	NA	3.1–5.7	2.9–5.2	4.2–6.4
Phosphorus	mmol/L	0.73–1.45	NA	NA	0.7–1.5	1.0–1.4
Iron	µmol/L	5.5–30.6	NA	NA	NA	9–27
Amylase	U/L	32–139	NA	38–163	43–164	60–180
LDH	U/L	223–681	NA	126–264	127–264	100–190

**Table 7:** Analytical precision for haematology assays.

Analyte	Between-Run Precision			ABX Precision
	Mean	SD	CV (%)	CV (%)
Haemoglobin	13.4	0.27	2.02	3.33
Haematocrit	38.3	1.02	2.65	2.47
Red Blood Cell Count (RBC)	4.73	0.09	1.91	1.24
Platelets	281	17.75	6.31	10.1
White Blood Cell, Total (WBC)	7.4	0.19	2.64	1.9

**Table 8:** Methods and analytical precision for clinical chemistry assays.

Analyte	Method	Between-Run Precision			Producer's Precision*	
		Mean	SD	CV (%)	Mean	CV (%)
Alanine Aminotransterase (ALT)	IFCC Modified without pyridoxal phosphate	44.5	1.5	3.5	57	4.6
Aspartate aminotransterase (AST)	IFCC Modified without pyridoxal phosphate	49.5	3.0	6.0	46	6.3
Alkaline Phosphatase (ALP)	*p*-Nitrophenyl phosphate. Diethanolamine	160.3	7.4	4.6	33	5.1
Amylase	2-chloro-4-nitrophenyl-α-maltotrioside	56.3	1.6	2.8	92	2.5
Creatine kinase, Total	IFCC, UV Kinetic/Imidazole Buffer	149.4	9.1	6.1	49	4.4
Gamma glutamyltransferase (GGT)	L-γ-Glutamyl-3-carboxy-*p*-nitroanilide	36.4	1.4	3.9	28	4.5
Lactate dehydrogenase (LDH)	UV kinetic (Pyruvate to Lactate)	309	10.8	3.5	333	3.9
Protein, Total	Biuret/endpoint	66.7	2.4	3.7	38	4.7
Albumin	Bromocresol green- Succinate Buffer	46.4	1.3	2.7	27	2.6
Bilirubin, Total	Malloy-Evelyn modified. End point	24.8	0.9	3.7	16.9	3.3
Bilirubin, Direct	Malloy-Evelyn modified. End point	10.1	0.7	7.2	10.9	3.5
Cholesterol	Cholesterol Oxidase/Peroxidase	2.2	0.1	4.0	3.3	3.8
Glucose	Glucose oxidase/Peroxidase	5.3	0.2	4.6	5.1	3.5
Iron	Chromazurol	22.0	1.1	4.9	19.9	5.4
Triglyceride	Lipase/GK/GPO/Peroxidase/dye	1.1	0.1	4.3	1.3	4.3
Urea	Enzymatic –UV Kinetic	7.6	0.4	5.4	9.8	4.5
Creatinine	Jaffé-Kinetic	96.1	3.8	3.9	136	4.9
Uric acid	Uricase/Peroxidase	275	12.5	4.6	286	1.8
Chloride	ISE, Direct	107.4	1.4	1.3	NP	2.0
Phosphorus	Phosphomolybdate formation	1.3	0.1	4.2	1.44	2.8
Potassium	ISE, Direct	4.7	0.1	2.9	NP	3.0
Sodium	ISE, Direct	148	2.0	1.3	NP	2.0

SD = Standard Deviation; CV = Coefficient of variation; NP = Not provided.

* Chloride, Potassium and Sodium by Human Diagnostics, Germany; Others by Elitech Diagnostics, France.

## Discussion

This study aimed at establishing haematological and biochemical reference values to serve as standards for the interpretation of laboratory results during screening and follow-ups in clinical trials and routine healthcare in the Kintampo area. The results obtained from the Kintampo area demonstrated that the red blood cell parameters (haemoglobin, haematocrit and RBC counts) were lower than values set as standards on the clinical haematology machines being used for clinical trials assessments in the study area. Such variations are expected for populations in different geographical/ecological locations; the recommendations of the manufacturers for each laboratory to establish its own reference values based upon the local population [13] has been proved beneficial. Values in the manual accompanying the haematology analyzer were defined using a population in New Jersey, USA. It is of interest to document also that the values obtained from our study were on most occasions far lower than those of other western industrialized countries [14], [15]. Similar observations have been made in studies carried out in Mampong Akuapem in southern Ghana [1]; Kericho in Kenya [16]; in southern and eastern Africa [7]; in Saudi Arabia [17], Erzurum; in Turkey [18] as well as Pakistan [19]. These lower values for areas in sub-Saharan Africa have been attributed to factors such as poor nutritional status, genetic red blood cell disorders (such as sickle cell trait) or parasitic infections including schistosomiasis or malaria [7]. No differences were observed in both haemoglobin and haematocrit values obtained in this study and that of the populations of southern Ghana [1]. A similar survey in the northern part of Ghana will help inform the scientific community about how much generalization one can make using the Kintampo area study results. The haemoglobin levels were, however, higher in this survey than those obtained from other populations in Kericho, Kenya [16].

Significant gender differences were documented for the RBC parameters (haemoglobin, haematocrit and RBC), and this is consistent with an already-established knowledge that males have higher values for these parameters than females [6], [15], [18], [20], [21]. The reasons for these differences have been attributed to factors such as the influence of the androgen hormone on erythropoiesis and menstrual blood loss in females [16], [21]. The demonstration of significantly higher platelet values in females than males supports findings from previous studies [1], [15], [16]. Platelet and WBC values documented in this study were generally lower compared with the values in the instrument manual as well as values reported by Kratz *et al.* [14] in the US and Wakeman *et al.* [15] in the UK. The values from this study were however similar to those reported in southern Ghana [1] and many other African countries [21], [22], [23], [24]. The cause(s) of lower platelet counts in Africans is not known [6]; however, the lower platelet values in our studies could be due to genetic factors [25] or increased consumption of platelets as a result of malaria infection in our study areas [26].

The most commonly requested haematology parameters for screening/enrolment of participants and monitoring safety during clinical trials are haemoglobin, haematocrit, total WBC and platelet counts [27], [28], [29]. The proportion of OOR values for these four parameters ranged between 16.1% and 53%. This means based on the previous reference values used in the study area, up to 53% of potential study participants would have been declared as having abnormal results or enrolled participants would be reported as having adverse events (AEs). In the area of clinical management of patients, a patient requiring a particular treatment may be denied it whiles one who does not need treatment would end up being treated due to the use of inappropriate reference values. In other studies, the OOR values for these parameters were up to 29% [7] and 44% [20] when the locally derived values were compared with US data.

Findings of significantly higher values in males than females for the following biochemical parameters (ALT, AST, ALP, Bilirubin, CK, GGT, iron, creatinine, urea), and vice versa for uric acid is generally supported by other studies [1], [16], [20] as shown in Table 6. The urea levels were low when compared to the values from the reagent inserts of Elitech Diagnostics and the US values [14]. However the results from this study were comparable to those from other African countries [16], [20] and from Saudi nationals [17].

Biochemical tests commonly used during screening/enrollment and safety monitoring of trial participants in the Kintampo study area are ALT, AST, Bilirubin (Total and Direct), Urea and Creatinine. The proportion of OOR values for these parameters was up to 32% in our study, compared to up to 42% in Kenya [7] and up to 81% in Tanzania [20]. The concern is about the levels of disqualification from screening/enrolment into clinical trials and mis-interpretations of AEs. Using the western values, we will report laboratory AEs in essentially normal volunteers, with the potential to ruin a trial (based on AEs) where there is no problem as such. Similar findings of higher ALT and AST values have been reported in south India [30]. Although the definite cause of higher liver enzymes in our population is unknown, there is the possibility of this being due to subclinical viral infections or the levels of usage of herbal preparations as discussed in earlier publications [1]. Screening for Hepatitis viruses was not performed in this study. However, published data on prevalence of these viruses among Ghanaian blood donors is 7 to 15% for Hepatitis B virus [31], [32] and 7 to 11% for Hepatitis C virus [31]. On the use of herbal preparations, it has been estimated that the first line treatment for 60% of children with fever resulting from malaria in Ghana, Mali, Nigeria and Zambia is the use of herbal medicine at home [33].

Between-run test measures a method's overall precision as it measures the random error inherent in the method from day to day. It takes into account variable factors such as changes in reagents, operators and ambient operating conditions. Between-run precision for haemoglobin and platelets were within precision limits quoted in the analyser manual. Although the CVs for the other analytes were higher (i.e. haematocrit 2.65 against 2.47%, RBC 1.91 against 1.24% and WBC 2.64 against 1.9%), they were within the Clinical Laboratory Improvement Act (CLIA) acceptable test performance criteria [34]. These 5 parameters are presented for haematology because they are the measured analytes from which the others are derived. Majority of the clinical chemistry tests were also within the precision limits indicated by the reagent producers. The precision of all the analytes were within the CLIA acceptable performance criteria limits. This precision data supports the reliability of the reference values established by this study.

### Conclusion

The reference values developed for the Kintampo study area will be of immense benefit to most clinical trials requiring monitoring of haematological and biochemical parameters and patient care in general. Compared to other references, the reference values for haemoglobin, haematocrit, red blood cell counts and urea are lower in the Kintampo study area.
